# Paralytic squint due to abducens nerve palsy : a rare consequence of dengue fever

**DOI:** 10.1186/1471-2334-12-156

**Published:** 2012-07-16

**Authors:** Mitrakrishnan C Shivanthan, Eranda C Ratnayake, Bandula C Wijesiriwardena, Kalum C Somaratna, Lakmal KGK Gamagedara

**Affiliations:** 1 Wards 45 & 46 A, National Hospital of Sri Lanka, Regent Street, Hospital Square, Colombo, Sri Lanka

**Keywords:** Dengue fever, Squint, Abducens palsy

## Abstract

**Background:**

Dengue fever is an endemic illness in the tropics with early and post infectious complications affecting multiple systems. Though neurological sequelae including mononeuropathy, encephalopathy, transverse myelitis, polyradiculopathy, Guillain-Barre syndrome , optic neuropathy and oculomotor neuropathy have been reported in medical literature, the abducens nerve despite its notoriety in cranial neuropathies in a multitude of condition due to its long intracranial course had not been to date reported to manifest with lateral rectus paralysis following dengue.

**Case presentation:**

A previously well 29 year old male with serologically confirmed dengue hemorrhagic fever developed symptomatic right lateral rectus palsy during the critical phase of the illness, which persisted into convalescence and post convalescence with proven deficit on Hess screen. Alternate etiologies were excluded by imaging, serology and electrophysiology.

**Conclusions:**

The authors detail the first reported case of abducens nerve palsy complicating dengue fever in a previously healthy male from Sri Lanka**.** In a tropical country with endemic dengue infections, dengue related abducens neuropathy may be considered as a differential diagnosis in cases of acquired lateral rectus palsy after dengue fever.

## Background

Dengue virus infections are known to manifest in three main forms: — classic dengue fever (DF, dengue haemorrhagic fever (DHF) and dengue shock syndrome (DSS) [1]. Morbidity and mortality can be minimized if accepted resuscitation protocols are adhered to and recovery is usually without residual disability. Neurological deficits complicating dengue fever has gained attention with the wide availability and use of diagnostic tools to diagnose dengue fever with certainty. Though mononeuropathy, encephalopathy, transverse myelitis, polyradiculopathy, Guillain-Barre syndrome, oculomotor neuropathy and optic neuropathy [2-7] have all been documented in literature to date, abducens nerve palsy has eluded clinical observation. The authors report the first case of a patient who developed a convergent paralytic squint due to right abducens palsy during the critical phase of dengue fever.

## Case presentation

A 29 year old male from Piliyandala, Sri Lanka was admitted with a four day history of high fever, arthralgia and myalgia during the mid 2011 epidemic of dengue fever. He had no eye complaints or other neurological symptoms on admission. There were no bleeding manifestations and urine output was satisfactory. He had no significant past medical history. His physical examination was unremarkable on admission apart from a body temperature of 39 degrees Celsius. His full blood count revealed a total white blood cell count of 7000/mm^3^ with 59% lymphocytes, hemoglobin 17.1 g/dl, PCV 51%, platelet count of 82,000/mm^3^ and elevated liver transaminases - ALT 83U/l and AST 82 U/l. The platelet count dropped to a nadir of 40,000/mm^3^ before convalescence. However the patient did not develop any clinical features of significant plasma leakage, shock or bleeding. The chest X-ray was normal. Dengue infection was confirmed with positive Dengue IgM antibodies capture enzyme-linked immunosorbent assay (MAC-ELISA). He was managed with intravenous fluids according to 2009 WHO dengue guidelines. On the 3^rd^ day at hospital he complained of new onset binocular diplopia which worsened on right gaze. Comprehensive neurological assessment failed to detect any other abnormality except for the convergent squint with right sided lateral rectus palsy causing diplopia maximal on gazing to right without fatiguability or nystagmus. All other eye movements, both optic fundi, visual acuity and colour perception were all unremarkable. The fever settled on day 8 of the illness and subsequently the platelet counts rapidly rose to normal levels. However the diplopia persisted.

**Figure 1:**
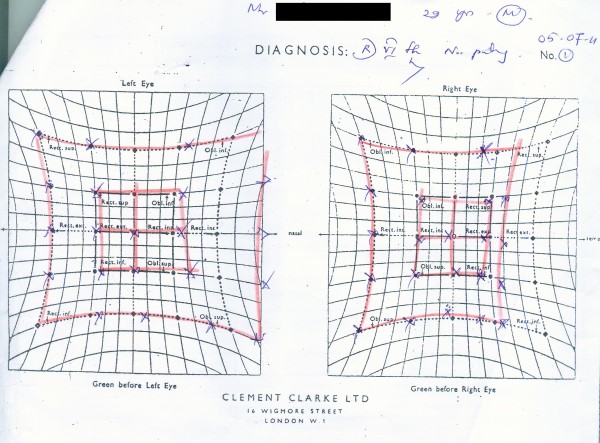
**Hess screen showing right 6**^**th **^**nerve palsy.**

Hess screen confirmed paralysis of right lateral rectus (Figure 1). Contrast enhanced computed tomography (CECT) and a subsequent MRI of the brain with thin orbital slices were unremarkable. Erythrocyte sedimentation rate was 10 mm in the 1^st^ hour. Serology was negative for anti nuclear antibodies, ANCA, HIV antibodies, Japanese Encephalitis IgM antibodies, Herpes simplex antibodies and Varicella antibodies. C3 and C4 complement levels were within normal limits. Cerebrospinal fluid studies were unremarkable. Nerve conduction studies and electromyography including repetitive nerve stimulation of limb muscles and orbicularis oculi were normal. He was managed expectantly for the abducens nerve palsy and reassured and reviewed monthly without pharmacotherapy. The abducens palsy which was still persistent on clinical evaluation at 3 weeks post discharge had resolved completely when the patient presented after 3 months subsequent to two defaulted visits to obtain his medical certificate.

## Discussion

Dengue infection is an endemic arboviral disease in the tropics and it often reaches epidemic proportion in Sri Lanka after the monsoon in June-July each year. Though dengue fever (DF, dengue haemorrhagic fever (DHF), and dengue shock syndrome (DSS) were the three main clinical presentations of dengue infection that broadly guided treatment by clinicians, a recent practical approach of classifying dengue as either without or with warning sings and severe dengue based on basic clinical and laboratory parameters is gaining popularity due to its practicality in patient management [1]. Although the clinical spectrum of the disease is well recognized, on occasion rare complications of the infection are encountered. Of the neurological manifestation of the disease, encephalopathy and Guillain-Barre syndrome are the commonest [2,3]. Radiculopathies, plexopathies and mononeuropathies as a complication of dengue fever are recognized phenomena [4-6]. However neuro-ophthalmological complications following dengue fever are rare. Optic neuropathy [7] and oculomotor nerve palsy [8] have been reported in literature previously following dengue fever but there is no precedence in literature of abducens nerve palsy. The pathophysiology behind these manifestation remains obscure due to paucity of literature but an immune mediated mechanism is suggested.

Electrophysiological study findings, exclusion of compressive mass lesions by imaging, clinical and serological exclusion of alterative infectious, inflammatory and vasculitic causes and the clear temporal relationship with dengue fever establish the relationship between abducens nerve palsy and dengue fever in our patient.

Mononeuropathies following dengue have been associated with demyelinating type of conduction defects with axonal components on nerve conduction studies [3,6]. Though the predominant mechanism in dengue encepahalitis and myelitis appear to be infective, the proposed mechanism behind radiculoneuropathies and Guillain Barre Syndrome are more in favour of immune mediated demyelination and axonal involvement [9]. Although a diagnosis of abducens nerve palsy due to dengue infection was made there is no established treatment for the established mononeuropathies following dengue fever. IV immunoglobulin and steroid have been tried in isolated cases of mononeuropathies [10]. Expectant management has also been shown to have an acceptable outcome in case reports [6] including a patient managed for diaphragmatic palsy in our infectious disease unit [11]. Our patient was managed expectantly and planned for periodic review to assess improvement with clinical resolution at three months.

With dengue fever reaching epidemic proportions in temperate countries like Sri Lanka, unusual complications of this very common disease are more likely to be seen, and the authors report this rare case of abducens nerve palsy as a complication of dengue fever and highlight the importance of therapeutic trials to assess the value of different modalities of treatment of neuropathies following dengue infection.

## Conclusions

This is the first reported isolated case of abducens neuropathy in a patient who had dengue infection. This case illustrates that post-dengue infected patients may develop abducens neuropathy as an isolated neurological complication. In a tropical country with endemic dengue infections, post dengue infection abducens neuropathy may be considered as a differential diagnosis in cases of acquired convergent squint with diplopia in compatible clinical scenarios.

## Consent

Written informed consent was obtained from the patient for publication of this case report and any accompanying images. A copy of the written consent is available for review by the Editor-in-Chief of this journal.

## Competing interests

The author(s) declare that they have no competing interests.

## Authors’ contributions

MCS was instrumental in evaluating the patient and drafting of this manuscript. BCW supervised the clinical management of the patient and preparation of this manuscript. ECR, CKS and KGKLG were instrumental in literature review and follow up of the patient. All authors read and approved the final manuscript.

## Pre-publication history

The pre-publication history for this paper can be accessed here:

http://www.biomedcentral.com/1471-2334/12/156/prepub
